# PPI-Diff: De Novo Generation of Peptide Binders via Resolution-Aware Geometric Diffusion

**DOI:** 10.3390/biom16040528

**Published:** 2026-04-01

**Authors:** Benzhi Dong, Sijia Li, Chang Hou, Dali Xu

**Affiliations:** School of Computer Science and Artificial Intelligence, Northeast Forestry University, Harbin 150040, China; nefudbz@nefu.edu.cn (B.D.); 2023122574@nefu.edu.cn (S.L.); houchang@nefu.edu.cn (C.H.)

**Keywords:** diffusion models, peptide drug design, de novo design, resolution-aware, protein protein interactions

## Abstract

Peptide binders, serving as a critical drug modality bridging small-molecule compounds and protein macromolecules, can effectively mimic the secondary structural elements of natural proteins. Peptides exhibit unique physicochemical advantages when targeting protein protein interaction (PPI) interfaces, which are typically characterized by flat surfaces and extensive contact areas. Recently, diffusion models represented by RFdiffusion have established a new computational paradigm for protein backbone generation by defining a denoising process over the rigid-body transformation group. However, in the de novo design of binders targeting “undruggable” PPI targets, this general paradigm encounters significant adaptability bottlenecks. First, its underlying rigid-body assumption struggles to accurately describe the dynamic induced-fit process of peptides at the binding interface. Second, it lacks sufficient robustness to the experimental resolution heterogeneity inherent in training data. Furthermore, the decoupled two-stage generation of sequence and structure severs the synergy of physicochemical properties, leading to backbones with idealized, singular secondary structures that lack authentic spatial binding capacity and reasonable side-chain physicochemical features. To address these challenges, this study proposes PPI-Diff, a novel generative framework. While preserving the generative capability of diffusion models, PPI-Diff introduces three core mechanisms: (1) a resolution-aware constraint mechanism that maps the measurement precision of experimental data into explicit contextual constraints to dynamically suppress geometric noise from low-resolution samples; (2) an internal-coordinate-driven manifold diffusion model that performs conformational evolution on a Riemannian manifold constructed by dihedral angles, balancing local stereochemical validity with the precise capture of flexible peptide conformations; and (3) a geometry-semantic synergistic modeling mechanism that leverages the evolutionary embeddings of a pre-trained protein language model (ESM-2) as latent variables to align structure generation with biophysical functions. Systematic benchmarking demonstrates that, on a strictly non-homologous test set, the binders generated by PPI-Diff significantly outperform existing baseline models in terms of interface contact density, stereochemical validity, and sequence novelty.

## 1. Introduction

In the landscape of modern drug discovery, peptides have emerged as a highly significant therapeutic modality, complementing traditional small-molecule chemical drugs and monoclonal antibody biologics [1]. Their unique molecular size and spatial topology allow them to effectively bridge the chemical space gap left by traditional therapeutics. Peptides combine the excellent tissue- and cell-penetrating capabilities of small molecules with the high target specificity and low off-target toxicity typical of antibodies, thereby serving as crucial molecular vectors bridging the microscopic chemical space and macroscopic biomacromolecules [2,3]. Particularly in targeting intricate protein protein interaction (PPI) networks, peptide molecules have demonstrated irreplaceable therapeutic potential [4]. According to statistics from structural and systems biology, the human interactome harbors over 650,000 potential PPI interfaces [5]. These interaction networks precisely regulate almost all essential biological processes, including cell proliferation and signal transduction [6].

However, drug development against such targets has long been hindered by their distinct biophysical and topological features. Unlike traditional targets with well-defined active sites, such as kinases or G protein-coupled receptors, PPI interfaces typically exhibit massive solvent-accessible surface areas (1500–3000 Å^2^) and are relatively flat, highly solvent-exposed, and devoid of the deep hydrophobic pockets upon which traditional small-molecule drugs heavily rely for stable binding [7]. Small-molecule drug discovery strategies based on the traditional “lock-and-key” model and high-throughput screening struggle to achieve sufficient binding free energy on these flat interfaces lacking geometric constraints, resulting in a large number of critical PPI targets being historically classified as “undruggable” [8]. In contrast, peptide molecules, with their longer amino acid backbones and diverse side-chain groups, can flexibly mimic secondary structural motifs of natural proteins, such as α-helices or β-sheets, enabling high-affinity recognition of flat PPI interfaces through extensive surface geometric complementarity [9].

From a molecular thermodynamic perspective, the targeting of PPI interfaces by peptides is intrinsically a physicochemical process involving complex energy compensation. According to the Gibbs free energy equation (Δ*G* = Δ*H* − *T*Δ*S*), free peptides in solution typically adopt highly dynamic conformational ensembles, possessing significant conformational entropy (S). When a peptide binds to a target, its conformation is forcibly restricted to a specific three-dimensional space, incurring an unavoidable and massive entropic penalty. For the binding process to proceed spontaneously (Δ*G* < 0), sufficient enthalpic compensation (Δ*H*) must be provided through the formation of an extremely dense non-covalent interaction network at the interface (e.g., hydrogen bond networks, salt bridges, aromatic stacking, and hydrophobic desolvation effects). However, conventional computational screening methods based on rigid-body docking often overestimate the binding enthalpy while severely underestimating the conformational entropy loss during binding. This thermodynamic computational bias is a major contributing factor to the significant reduction in affinity frequently observed in many computer-designed peptide sequences during in vitro validation. Therefore, developing a novel computational framework capable of precisely simulating the dynamic conformational evolution of peptides and implicitly capturing thermodynamic equilibrium has become an urgent priority to overcome the bottleneck of PPI target druggability. Although peptide therapeutics exhibit significant physicochemical advantages in targeting PPI interfaces, their discovery process remains severely constrained by the exploratory capacity of current experimental techniques. The potential sequence space of peptides grows exponentially with amino acid chain length [10]. Mainstream experimental technologies, such as phage display or combinatorial chemical libraries, can only perform highly sparse and locally biased sampling within this enormously multidimensional chemical space [11]. To thoroughly overcome the limitations of biological experiments in the face of combinatorial explosion, the foundational paradigm of peptide drug research is undergoing a fundamental shift from traditional “trial-and-error screening” to data-driven de novo design based on first principles [12]. Early computational protein design primarily relied on heuristic search algorithms governed by physical energy force fields (e.g., the Rosetta modeling framework) [13]. However, due to the extremely rugged energy landscape of protein folding and the vast number of local optima, traditional physical methods often face exponential surges in computational complexity when designing complex peptide binders. Recently, breakthrough advancements in geometric deep learning and generative artificial intelligence have dramatically propelled the methodological evolution of biomacromolecular computational design [14,15,16]. In the domain of sequence-structure co-design, several representative computational paradigms have emerged. For instance, ProteinMPNN exhibits outstanding sequence decoding and amino acid design capabilities given a fixed backbone [17]; however, as a purely inverse folding model, it is strictly dependent on fixed backbone inputs, completely lacking the ability to autonomously explore the peptide conformational space. Meanwhile, diffusion models such as FrameDiff and Chroma have further expanded the geometric constraint boundaries of backbone generation [18,19], yet they remain insufficient in addressing the stringent side-chain physicochemical matching required by complex PPI interfaces. Among numerous generative architectures, the RFdiffusion model has successfully solved the inverse physical problem of progressively recovering protein backbone topologies from random Gaussian noise by rigorously defining a Markovian diffusion process over the 3D Euclidean space and the SE(3) rigid-body transformation group [20]. This model has demonstrated excellent computational robustness in general tasks such as monomeric globular protein generation and specific enzyme active-site scaffolding, establishing the current performance baseline for rigid-body-assumption-based structure generation. This study selects RFdiffusion as the core evaluation and benchmarking baseline to investigate the specific theoretical and practical limitations faced by this general generative paradigm in depth when directly transferred to the highly flexible PPI peptide binder design task. First, there is a physical conflict between the rigid-body assumption and the intrinsic flexibility of peptides. The core algorithm of RFdiffusion abstracts amino acid residues as non-deformable rigid geometric units [21]. While this assumption is rational for compactly folded globular proteins, unbound peptides typically exhibit properties of intrinsically disordered proteins (IDPs), and their binding process involves significant conformational adjustments (induced-fit effects) [22]. General models based on rigid-body assumptions tend to generate the lowest-energy straight, rigid helical structures, struggling to adapt to the complex topologies of target surfaces through flexible backbone bending. Second, error accumulation in geometric features is triggered by data resolution heterogeneity. Existing training strategies treat all structural data in the Protein Data Bank (PDB) equally. In the absence of an explicit resolution-aware mechanism, the model inevitably averages the spatial features of data with varying qualities during feature extraction, thereby blurring the fine side-chain packing geometries essential for high-affinity binding [23]. Third, the decoupling of geometric generation and biological semantic sequencing leads to a lack of physicochemical features. The two-stage separated design paradigm leaves the geometric generation model in a “sequence-blind” state, rendering it unable to foresee the spatial steric hindrance and physicochemical repulsion introduced by bulky side-chain groups. Consequently, the generated backbones are difficult to pack reasonably with real sequences, significantly reducing their folding stability in physical environments. To address these prevalent theoretical challenges, this study proposes PPI-Diff, a de novo peptide design framework that deeply integrates low-level physical awareness with high-level semantic enhancement. This framework performs a foundational physical reconstruction of existing generative paradigms through three core mechanisms: (1) A resolution-aware condition injection mechanism maps the resolution of experimental data into continuous conditional embedding vectors to dynamically reweight the training loss, guiding the model to prioritize high-fidelity structural features and actively filter geometric noise from low-quality data. (2) An internal-coordinate manifold diffusion mechanism constructs a diffusion process on a Riemannian manifold formed by dihedral angles to more efficiently capture the dynamic flexible conformational transitions of peptides at the binding interface. (3) A geometry-semantic synergistic generation mechanism integrates the evolutionary representations of the pre-trained protein language model (ESM-2) [24] as latent variable conditions, achieving end-to-end joint optimization of structure and sequence.

## 2. Materials and Methods

### 2.1. Overall Model Architecture

**Figure 1:**
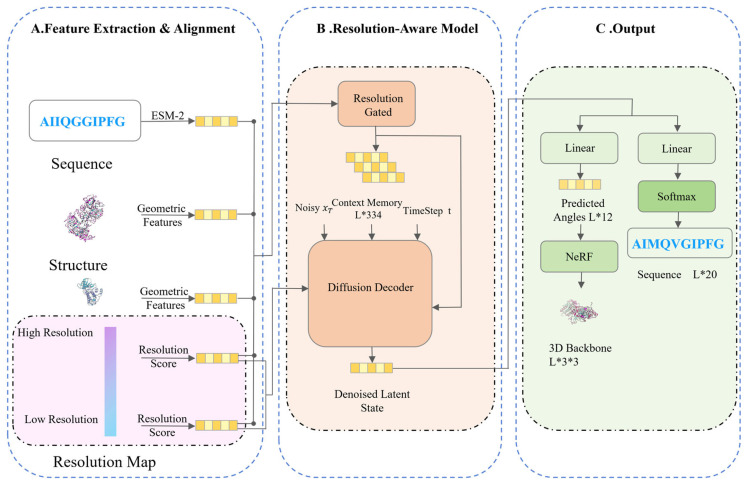
Overall architecture of the PPI-Diff model. (**A**) Feature Extraction & Alignment stage, where target geometric features and ESM-2 sequence embeddings are extracted. (**B**) Transitioning to the Resolution-Aware Model; these multimodal features are fused with inverse-proportional resolution scores to form a context memory, which dynamically guides the diffusion decoder during the reverse denoising steps. (**C**) Finally, in the Output stage, the denoised latent states are simultaneously decoded into 3D continuous backbone coordinates via NeRF and discrete amino acid sequences via a linear projection, achieving synergistic generation. The notation L*3*3 denotes the tensor dimensions, where L is the sequence length, 3 represents the backbone atoms (N, Cα, C), and 3 represents the spatial coordinates (x, y, z). Solid arrows indicate the direction of data flow, and dashed lines represent the boundaries of functional modules. Different colors are used to distinguish distinct data processing stages.

This study proposes PPI-Diff, a conditional diffusion model incorporating physical constraints. This architecture utilizes experimental resolution as explicit regulatory information to dynamically guide the denoising generation process in high-dimensional space. The overall computational framework of PPI-Diff encompasses three progressively advanced stages of data processing and feature generation (Figure 1). The first stage is data preprocessing and weighted fusion, which suppresses coordinate distortion interference from low-quality flexible regions at the source. The second stage is resolution-aware denoising, where the resolution map is explicitly encoded into a high-dimensional confidence tensor and injected into the decoder. The third stage is the synergistic generation of structure and sequence in the latent space, performing joint gradient optimization within the same latent variable space.

### 2.2. Data Preprocessing and Resolution-Aware Mechanism

High-quality 3D structural data is the core guarantee for the network to accurately learn physical interaction laws. In the initial stage of dataset construction, high-confidence protein–protein interactions (combined score ≥ 400) were carefully curated from the STRING database (version 12.0) [25]. When mapping these high-confidence interactions to 3D structures in the PDB, 94.2% of the interaction pairs were successfully aligned and extracted as high-quality, single-chain conformations for both the target and binder proteins via the UniProt interface [26]. Interactions lacking complete or reliable structural data were rigorously discarded. This strict structural filtering pipeline ultimately yielded a highly refined subset of 2850 unique PPI pairs. Furthermore, to handle complex multi-chain PDB files, our pipeline did not rely on blind distance-based cropping. Instead, we precisely extracted the specific interactive chains corresponding to the unique UniProt IDs provided by STRING, effectively decoupling the target-binder pairs from irrelevant auxiliary chains or co-factors. During the training phase, bidirectional physical symmetry augmentation was applied to these pairs, yielding 5700 effective structural samples for the network.

To ensure the rigor of the model evaluation and completely eliminate homology leakage between the training and test sets, this study introduced a strict sequence redundancy removal process. Using the CD-HIT clustering algorithm (version 4.8.1) [27], the sequence identity threshold was strictly set to 30%. The similarity between all protein sequences in the test set and the training set samples was forcibly restricted to below 30% (i.e., the universally recognized “twilight zone” in structural biology [28]). This strict data partitioning fundamentally eliminates the possibility of sequence memorization, ensuring that the generative capabilities demonstrated by the PPI-Diff model stem from a profound internalization of underlying physical mapping rules.

**Figure 2:**
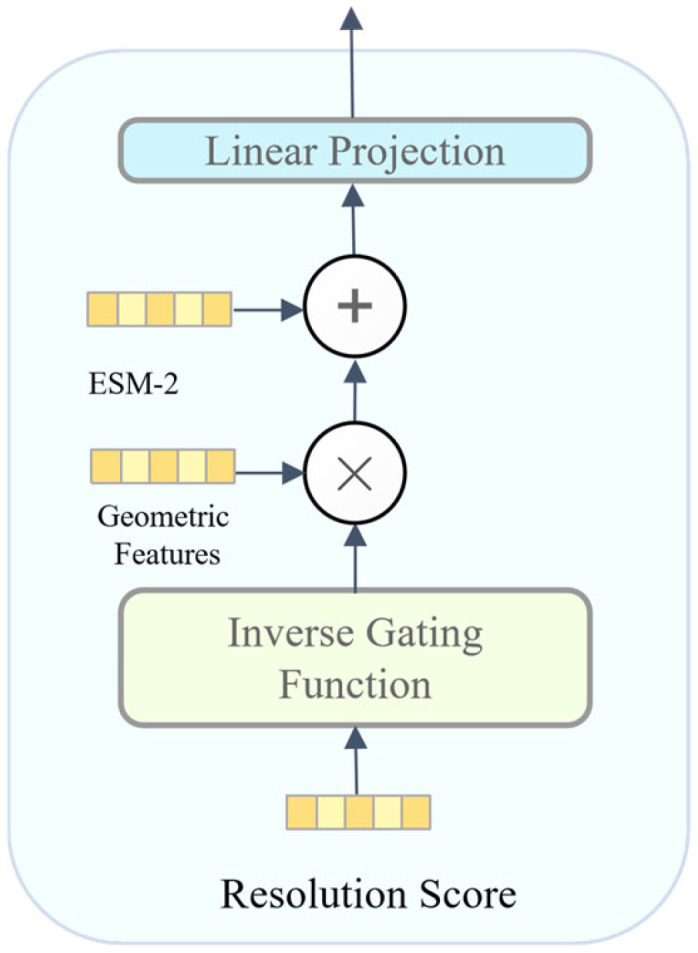
Detailed schematic of the resolution gating mechanism and multimodal feature fusion. Solid arrows indicate the direction of data flow. Different colors are used to visually distinguish the various input modalities (e.g., sequence, structure, resolution) and processing modules.

Experimentally resolved structures of real target proteins often contain flexible regions with extremely poor local resolution. Indiscriminately encoding such noise induces severe overfitting. A dedicated physics-aware feature representation system is designed at the front end of the architecture, receiving the target sequence, 3D coordinates, and resolution map $R\in R^{L_{\mathrm{tgt}}\times 1}$ as joint inputs. The local resolution value *R_i_* of target residue i is mapped to a reliability weight with inverse proportional monotonicity:

(1) $$w_{i}=\phi (R_{i})=\frac{1}{R_{i}+\epsilon }$$

where *ϵ* is a minute smoothing constant to prevent division-by-zero overflow during forward propagation. A parameter-free inverse-proportional function was chosen over exponential or sigmoidal alternatives to avoid introducing additional hyperparameters that could lead to overfitting on the training set’s specific resolution distribution. Based on this weight, element-wise gating signal modulation is applied to the initial high-dimensional geometric features ${F\;}_{\mathrm{geo}}^{\left(i\right)}$(Figure 2):

(2) $${F\;}_{\mathrm{geo}}^{\left(i\right)}={F\;}_{\mathrm{geo}}^{\left(i\right)}⊗\mathrm{Broadcast}(w_{i})\;$$

### 2.3. Multimodal Feature Fusion

In the multimodal semantic feature extraction stage, the pre-trained weights of the large-scale protein language model ESM-2 are frozen, serving solely as a feature extractor. For the input sequence, ESM-2 outputs a high-dimensional dense representation with dimensions $R^{L\times 1280}$ [24]. To reduce the VRAM load of the downstream denoising network and achieve modality alignment, a Linear Projection layer is employed to directly map this feature representation to the latent space dimension $R^{L\times 256}$. Simultaneously, the 3D geometric features of the target are also projected to 256 dimensions after a graph convolution operation with a kernel size of 3. The purified and filtered high-fidelity geometric features are then concatenated and fused with the deep evolutionary semantic representation $F_{\mathrm{seq}}$ extracted by ESM-2. The resulting context memory matrix *c*_target_ ultimately forms a high-fidelity tensor of $R^{L_{\mathrm{tgt}}\times 512}$, providing ample conditional capacity for the diffusion model:

(3) $$c_{\mathrm{target}}=\mathrm{Linear}(F_{\mathrm{geo}}⊕F_{\mathrm{seq}})$$

### 2.4. Internal-Coordinate Manifold Diffusion

The study employs an internal coordinate system to rigorously parameterize the kinematic backbone of the peptide. The complete conformational state $a_{i}$ of the *i*-th residue in the peptide chain is defined as a 6D angular vector comprising three backbone dihedral angles and three local bond angles [29]:

(4) $$a_{i}\;=\;[\phi_{i},\psi_{i},\omega_{i},\theta_{1,i},\theta_{2,i},\theta_{3,i}{]}^{T}$$

To resolve the gradient breakage caused by the periodicity of angular data on a topological torus, a continuous trigonometric feature embedding method is introduced to map the angles onto a 2D unit circle [30]:

(5) $$v_{\alpha }=[\cos\alpha ,\sin\alpha ]$$

In the conditional diffusion stage, the core decoder of the diffusion model discards the computationally redundant complex dual-stream architecture, opting instead for a more computationally efficient 4-layer deeply stacked Transformer Decoder module. Within each layer of this cross-attention architecture, the number of heads in the Multi-head Attention mechanism is set to 8, the total dimension of the model’s latent variables is 512 (with a single-head feature dimension of 64), and the expansion ratio of the hidden Feed-Forward Network (FFN) is set to 4. Pre-LayerNorm structures and GeLU activation functions are utilized to ensure the smoothness of nonlinear mappings and the stability of deep gradient propagation within the network [31].

To evade non-realistic conformations such as molecular bond breaks caused by redundant degrees of freedom in the 3D Cartesian coordinate system from the physical bottom layer, the PPI-Diff framework abandons traditional 3D spatial diffusion. Instead, it defines a conditional diffusion process on a continuous internal coordinate manifold constructed by peptide backbone dihedral angles and bond angles.

For a given initial angular feature $x_{0}\in R^{L\times D_{\mathrm{angle}}}$ of a native peptide ligand, the forward diffusion process is defined as a Markov chain with fixed parameters. This study adopts a Linear Schedule strategy. Here, the variance schedule parameter $\beta_{t}$ is explicitly defined as the factor controlling the scale of Gaussian noise added at each forward diffusion step. Specifically, $\beta_{t}$ linearly increases with the time step t from an initial value of $\beta_{\mathrm{start}}=0.001\;$ to a final value of $\beta_{\mathrm{end}}=0.02$. Based on the reparameterization trick of Denoising Diffusion Probabilistic Models (DDPM), defining $\alpha_{t}\;=\;1-\beta_{t}$ and its cumulative product form ${\bar{\alpha }}_{t}=\prod_{s=1}^{t}\alpha_{s}$, the noisy conformation *x_t_* at any arbitrary time step *t* can be obtained via closed-form sampling directly from the initial conformation:

(6) $$x_{t}=\sqrt{{\bar{\alpha }}_{t}}x_{0}+\sqrt{1-{\bar{\alpha }}_{t}}\epsilon$$

where ϵ∼N(0,1) is a random perturbation term following a standard normal distribution. This continuous noise-adding mechanism, based on a pure angular manifold, constitutes the physical foundation and training objective for the subsequent neural network to perform reverse denoising.

### 2.5. Cross-Attention Denoising and Synergistic Generation

**Figure 3:**
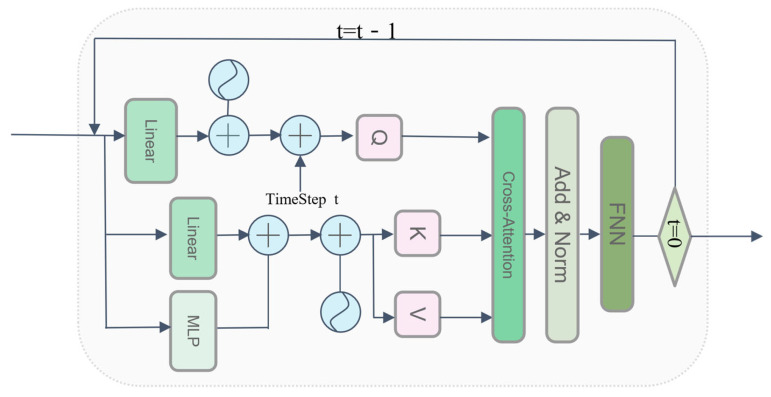
Schematic of the cross-attention architecture in the resolution-aware conditional diffusion model decoder. Solid arrows indicate data flow. The letters Q, K, and V stand for the Query, Key, and Value feature matrices in the attention mechanism, respectively. The symbol represents element-wise addition, and the diamond shape indicates the condition check for the final time step (t = 0).

In the reverse generation (denoising) stage, the network achieves deep physical coupling between the peptide conformational manifold and the target microenvironment through a core Cross-Attention mechanism (as shown in Figure 3). Specifically, the noisy peptide conformation $x_{t}$ at the current time step t, after undergoing linear projection, is sequentially injected with sinusoidal positional encodings and time-step embeddings to generate the Query vector (Q) for the attention mechanism. Concurrently, the target context memory features—fused via ESM-2 semantic extraction and geometric graph convolution—are first superimposed with the dynamic resolution tensor via a Multilayer Perceptron (MLP) for feature correction, subsequently injected with positional encodings, and ultimately projected into Key (K) and Value (V) vectors. Specifically, this feature correction is modulated via a two-layer MLP utilizing the SiLU (Swish) activation function, ensuring smooth gradient flow during the diffusion process. Furthermore, the feed-forward networks within the Transformer decoder blocks employ GELU activations.

In the Cross-Attention module, the peptide query vector Q, carrying spatio-temporal states, attends to and retrieves high-confidence geometric and semantic features from the target context K, V, precisely simulating the dynamic induced-fit effect of the ligand accommodating the complex target interface within the latent space. The interacted features sequentially pass through residual connections, layer normalization (Add & Norm), and the Feed-Forward Network (FFN), ultimately outputting the predicted denoising state *ϵ_θ_* for the current time step, and the conformational state is updated via the DDPM reverse sampling formula [16]:

(7) $$x_{t-1}\;=\;\frac{1}{\sqrt{\alpha_{t}}}\left(x_{t}-\frac{1-\alpha_{t}}{\sqrt{1{\bar{\alpha }}_{t}}}\epsilon_{\theta }(x_{t},t,c)\right)+\sigma_{t}z$$

In the overall design of the network architecture, the Sequence Decoder module and the structural diffusion module maintain a high degree of logical homology, running in parallel as independent branches. The sequence prediction network adopts a Transformer cross-attention architecture nearly identical to that in Figure 3, directly receiving the 3D angular features of the peptide conformation and the resolution-corrected target context features as joint inputs (due to its non-diffusion nature, this branch requires no time-step injection). Through self-attention and cross-attention mechanisms, the network can accurately predict the discrete probability distribution of amino acid types at specific spatial positions. This study achieves synergistic parameter updating in a unified computation graph by jointly optimizing the mean squared error loss of structural diffusion and the cross-entropy loss of sequence prediction during backpropagation [32]. This joint training strategy enables the network to autonomously internalize the bidirectional constraint laws of amino acid sequence and 3D topology within the latent space.

At the terminus of the generative inference stage for structural topology, this study introduces the Natural Extension Reference Frame (NeRF) algorithm [29] to autoregressively convert the predicted pure angular features into the 3D absolute spatial positions $r_{D}$ of backbone atoms:

(8) $$r_{D}\;=\;r_{C}\;+{\;l}_{\mathrm{CD}}\cdot R(r_{A},r_{B},r_{C})\cdot [\sin\theta \cos\tau ,\sin\theta \sin\tau ,\cos\theta {]}^{T}$$

where $r_{A},{\;r}_{B},\;r_{C}$ are the known coordinates of preceding atoms, ${\;l}_{CD}$ is the bond length, θ and τ are the bond angle and dihedral angle, respectively, and *R* is the rotation matrix. By explicitly incorporating rigid physical priors such as fixed peptide bond lengths and basal bond angles, this module strictly ensures from the mathematical bottom layer that the generated protein backbone structures highly conform to fundamental stereochemical rules. Importantly, unlike models that directly predict 3D Cartesian coordinates and require explicit chirality penalty terms, PPI-Diff operates entirely within the internal coordinate space. Through this NeRF reconstruction, standard bond lengths and planar angles are strictly preserved, inherently guaranteeing the correct L-amino acid stereochemistry (chirality) of the generated backbones without the need for auxiliary constraints.

### 2.6. Loss Function and Joint Optimization Strategy

To ensure stable convergence of the PPI-Diff framework within an ultra-high-dimensional parameter space, this study constructs a jointly optimized Composite Loss Function. The overall objective function $L_{\mathrm{total}}$ comprises the angular reconstruction loss of denoising diffusion and the sequence cross-entropy loss:

(9) $$L_{\mathrm{total}}\;=\;\lambda_{\mathrm{geo}}L_{\mathrm{geo}}\;+{\;\lambda }_{\mathrm{seq}}L_{\mathrm{seq}}$$

where the geometric structural loss ℒ_geo_ employs the standard denoising Mean Squared Error (MSE). This loss directly measures the deviation between the noise tensor $\epsilon_{\theta }$ predicted by the network and the actual Gaussian noise ϵ added to the peptide angular manifold, thereby optimizing the model’s reverse denoising capability on the continuous internal coordinate manifold:

(10) $$L_{\mathrm{geo}}\;=\;\mathrm{MSE}(\epsilon_{\theta }(x_{t},c,t),\epsilon )$$

The sequence semantic loss $L_{\mathrm{seq}}$ employs Cross-Entropy loss to guide the network in accurately predicting the discrete probability distribution of amino acids at each position, constrained by the given spatial geometry and target context features:

(11) $$L_{\mathrm{seq}}=-\sum_{i=1}^{L}\log p(y_{i}|x_{0},c)$$

In actual training dynamics, the model performs static weighted summation of the two losses via hyperparameter weights ($\lambda_{\mathrm{geo}}$ and $\lambda_{\mathrm{seq}}$) and executes backpropagation within a unified computation graph.

### 2.7. Experimental Environment

All deep learning computations, diffusion reverse sampling, and model training were deployed on a high-performance computing node equipped with two NVIDIA RTX 4090 GPUs (NVIDIA Corporation, Santa Clara, CA, USA). The operating system environment was Ubuntu 20.04 LTS, utilizing PyTorch 2.0 with a CUDA 11.8 acceleration backend. During the generative inference stage, the total time steps T for the Denoising Diffusion Probabilistic Model (DDPM) were set to 1000. To ensure the stability of Markov chain sampling, a Cosine Schedule was adopted for the noise variance schedule, whose smoothing properties at the boundaries effectively prevented gradient collapse during the late stages of highly flexible conformation generation.

## 3. Results

### 3.1. Implementation Details

In a realistic protein protein interaction (PPI) microenvironment, the physical binding between the target and the binder interfaces generally exhibits relative geometric and electrostatic complementarity, though local conformational changes can introduce asymmetry. However, traditional generative models often employ a unidirectional “target-to-binder” mapping strategy, which easily leads to severe unidirectional overfitting of the network to specific target topologies. To break this limitation and significantly enhance the model’s generalization capability on unseen targets, this study introduced a bidirectional physical symmetry data augmentation strategy during the training forward propagation stage. Specifically, for any native PPI complex pair (ProtA, ProtB) in the training set, the network not only learns to reverse-diffuse and generate ProtB using ProtA as the static context condition; it is also forced to reverse their physical roles, requiring the network to generate ProtA using ProtB as the context condition. Through this mechanism, the effective training data volume was directly doubled without introducing any external noise. This double symmetry augmentation in the 3D Cartesian coordinate system greatly expanded the phase space of the training data and, more fundamentally, forced the diffusion network to improve statistical robustness and generalization by internalizing the universal symmetric properties of interface interactions, thereby serving as a core mechanism for maintaining the model’s generalization ability on non-homologous test sets.

Because this study aims to evaluate the generative efficacy of an end-to-end single model, the initial poly-glycine backbones output by RFdiffusion were directly extracted for topological feature and spatial contact evaluation, without introducing additional third-party sequence design tools (such as ProteinMPNN). To ensure the strict fairness and rigor of the comparative experiments, the inference execution of the baseline model RFdiffusion (version 1.1.0) strictly followed the default configuration protocols of its official open-source repository. In the de novo generation task for PPI binders, the input preprocessing retained only the core spatial coordinates of the target protein, masking all sequence and structural information of the native ligands. After the baseline model generated the backbone structure, no manual side-chain reconstruction was performed; its default generated poly-glycine backbones and corresponding topological parameters were directly extracted for quantitative evaluation. To comprehensively and objectively quantify the design quality of peptide binders, this study constructed a multi-scale evaluation metric system from three independent dimensions: interface geometric complementarity, molecular topological morphology, and physicochemical properties. All microscopic structural metrics were calculated with high precision based on the 3D Cartesian absolute coordinates of the complexes.

(1) Interface Contacts

The number of interface contacts directly reflects the density of the non-covalent interaction network formed between the peptide and the target. The effective contacts Cint between the peptide ligand and the target protein are defined as the number of heavy atom pairs (i.e., non-hydrogen atoms) whose spatial Euclidean distance is less than a specific threshold:

(12) $$C_{int}=\sum_{i\in L}\sum_{j\in T}I(||r_{i}-r_{j}|{|}_{2}\leq d_{cutoff})$$

where L is the peptide sequence set, T is the target sequence set, *r_i_* and *r_j_* represent the 3D coordinates of the corresponding heavy atoms, and the indicator function I(⋅) equals 1 when the interatomic distance is less than or equal to the physical cutoff threshold *d*_cutoff_ (uniformly set to 4.5 Å), and 0 otherwise.

(2) Radius of Gyration (*R*g)

The radius of gyration is a core macromolecular physical quantity that measures the structural compactness and topological extension of the generated peptide in 3D space. For a peptide backbone containing *N* α-carbon atoms, its *R*g is defined as the root-mean-square distance of each atom’s mass relative to the molecule’s center of mass:

(13) $$R_{g}=\sqrt{\frac{1}{N}\sum_{i=1}^{N}(r_{i}-r_{\mathrm{center}}{)}^{2}}$$

where $r_{center}=\frac{1}{N}\sum_{i=1}^{N}r_{i}$ is the coordinate of the main-chain center of mass for the peptide. A smaller Rg corresponds to a densely folded globular conformation, whereas a larger Rg indicates a highly flexible extended linear or random coil state.

(3) Sequence Identity

To rigorously verify the generative model’s ability to explore novel chemical spaces and avoid data leakage, a global sequence alignment algorithm was employed to quantify the homology between the generated sequences and native ligands:

(14) $$\mathrm{Seq\_Id}=\frac{N_{match}}{\max(L_{pred},L_{nat})}\times 100\%$$

where $N_{match}$ is the number of perfectly matched identical amino acid residues after global alignment, and $L_{pred}$ and $L_{nat}$ are the absolute lengths of the generated sequence and the native sequence, respectively. Unlike traditional local alignment metrics, the denominator here utilizes $\max(L_{pred},L_{nat})$ to strictly penalize length discrepancies. This design ensures a rigorous evaluation of structural homology in de novo generation tasks, where the generated binder lengths may significantly deviate from native ligands. Furthermore, the pre-trained AlphaFold 3 network (version 3.0.0) was invoked to output the predicted Local Distance Difference Test (pLDDT) score for each residue to measure the local folding confidence and structural stability of the conformations [33]; the DSSP algorithm (implemented via Biopython version 1.81) was called to determine and calculate the average helical content (Helicity) of the backbones; and the ProtParam toolset (provided by the ExPASy server, accessed on 25 February 2026) was utilized to calculate the isoelectric point (pI) and the Grand Average of Hydropathicity (GRAVY) index of the macromolecules. These multi-dimensional physicochemical metrics collectively formed a rigorous evaluation closed loop.

### 3.2. Quantitative Evaluation of Binding Modes and Physical Space Adaptation

While the exact thermodynamic binding free energy (ΔG) requires resource-intensive molecular dynamics calculations, this study evaluates the implicit thermodynamic optimization of PPI-Diff through macroscopic conformational dynamics and geometric complementarity metrics. To comprehensively validate the advantages of the resolution-aware synergistic generative framework in overcoming the fragmentation of the two-stage design, this study conducted an in-depth comparative evaluation between PPI-Diff and the baseline model RFdiffusion. Unlike traditional literature that solely focuses on the pure geometric coordinate deviations of generated backbones, this study constructed an evaluation system more aligned with real PPI scenarios from three dimensions: conformational dynamics, interfacial binding force, and physicochemical consistency. The core quantitative evaluation data are summarized in Table 1.

To further objectively measure the generative models’ capability to explore the conformational space, deep statistical analyses were performed on all samples in the test set. The data revealed a severe mode collapse problem in the baseline model’s conformational search. At the secondary structure level, the median helicity of the molecules generated by RFdiffusion directly hit 1.00, meaning that over half of the samples were completely locked into a pure rigid straight-helix state. The standard deviation of its radius of gyration (*R*g) was merely 1.30 Å, suggesting that the baseline model exhibited severely restricted adaptive elasticity to complex topologies. In contrast, PPI-Diff effectively released conformational degrees of freedom through Riemannian manifold diffusion. Statistics showed that the median helicity of PPI-Diff dropped to 0.08, indicating a natural preference for generating highly flexible regions; simultaneously, its radius of gyration exhibited immense physical extension elasticity. This extremely high statistical variance conclusively demonstrated that PPI-Diff could adaptively generate morphologically diverse “dynamic mesh-like wrapping” structures based on target topographies. To further decouple the physical effect of chain length from conformational adaptability, a coupling analysis utilizing the Pearson correlation coefficient between peptide chain length and contact number demonstrated that RFdiffusion’s correlation coefficient was only 0.095, indicating the model merely generated ‘longer rigid sticks’. In contrast, under the PPI-Diff architecture, this coefficient significantly increased to 0.232, validating its superior adaptive elasticity. This end-to-end synergistic mechanism enables the model to effectively transform 1D chain resources into 3D contact efficacy by deeply adhering to the complex grooves of the target. Crucially, the concern regarding the dependence of $R_{g}$ on chain length was explicitly investigated. Our results reveal that the correlation between $R_{g}$ and peptide length in PPI-Diff is notably low (0.164), whereas RFdiffusion exhibits a much higher dependency (0.438). This quantitative decoupling confirms that PPI-Diff’s spatial occupancy is driven by the geometric complementarity of the interface rather than simplistic linear expansion with length, thereby demonstrating true ‘topological elasticity’.

In the evaluation of structural physical rationality, the number of atomic clashes in the conformations generated by PPI-Diff was 15.4, compared to 0 for RFdiffusion. This objective result reveals the balancing dilemma between maximizing geometric structural adaptation and adhering to rigorous physical constraints. RFdiffusion achieved zero clashes by introducing empirical physical energy terms, but at the cost of severely restricting its freedom to explore tightly bound conformations. This study posits that PPI-Diff’s moderate relaxation of strict steric repulsion constraints is an inevitable consequence of the model trading off for greater conformational search elasticity within the latent space. Therefore, the structures generated by PPI-Diff should not be viewed as final static complexes, but rather as highly promising Thermodynamic Initial Coordinates. These structures capture the global topological potential of the peptide deeply adhering to the target. In actual application pipelines, a brief molecular force field energy minimization (e.g., Rosetta FastRelax or OpenMM) or side-chain repacking can easily eliminate these microscopic clashes, transforming them into high-affinity physical steady states.

**Figure 4:**
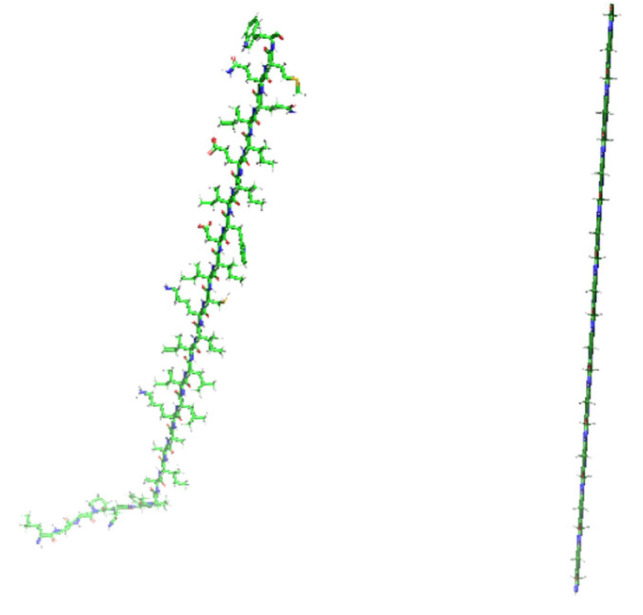
Spatial comparative validation of conformational topology and microscopic binding modes. Macroscopic 3D conformational comparison of binders generated by PPI-Diff (**left**) and the baseline model RFdiffusion (**right**). The baseline model exhibits a distinct preference for rigid straight helices, whereas PPI-Diff presents a flexible random coil state capable of accommodating complex target topologies. In the molecular representations, atoms are colored by element: carbon in green, nitrogen in blue, oxygen in red, and sulfur in yellow.

When dealing with complex targets lacking regular binding grooves (such as Q9C005) (as shown in Figure 4), RFdiffusion exhibited significant spatial mismatch, generating near-perfect rigid straight helices. Because the baseline model output a poly-glycine sequence devoid of side chains, it was entirely incapable of displacing water molecules to create desolvation effects, leading to a physical failure with zero effective contacts on the Q9C005 target.

**Figure 5:**
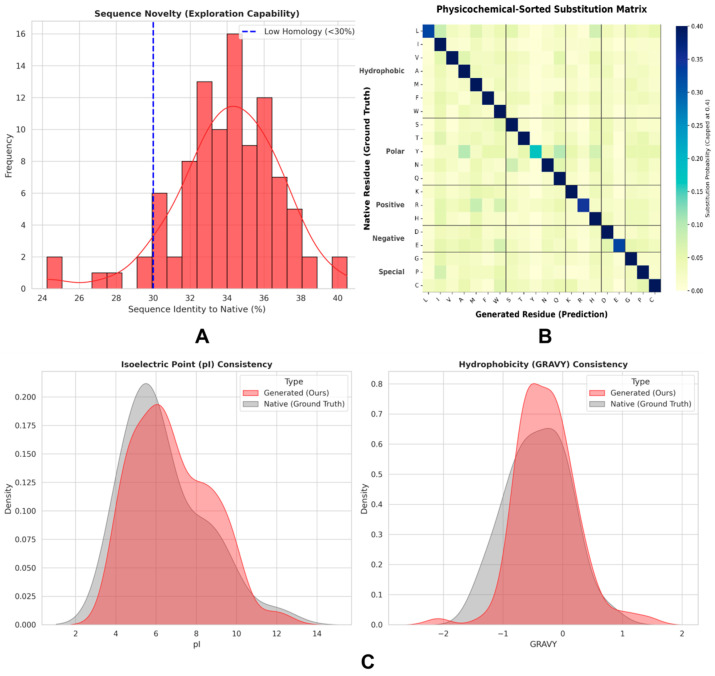
Analysis of homology crossing in sequence space and physicochemical property evolution. (**A**) Sequence identity distribution between generated sequences and native ligands. The blue dashed line indicates the 30% “twilight zone” threshold for structural homology modeling, demonstrating that the model achieves a high degree of non-memorized generation. (**B**) Residue substitution matrix sorted by physicochemical properties. High-probability substitutions are strictly concentrated within residue clusters sharing similar properties. (**C**) Probability density distributions of the isoelectric point (pI) and grand average of hydropathicity (GRAVY) index for generated binders and native ligands, confirming the robust conservation of macroscopic physicochemical features. In panel (**A**), the red solid line represents the fitted probability density curve of the sequence identity distribution. In panel (**B**), the solid black lines delineate the boundaries between distinct physicochemical categories of amino acids (Hydrophobic, Polar, Positive, Negative, and Special).

To verify the models’ exploratory capacity in sequence space, rigorous global alignment analyses were conducted between all generated peptide sequences and native ligand sequences. The results showed (Figure 5A) that the identity distribution between PPI-Diff-generated sequences and native ligands presented a standard unimodal pattern, with the Gaussian fitting mean stabilizing at 34.5%. Further full-sample statistics revealed an average similarity of only 5.4%, with a large number of samples successfully falling into the “twilight zone” of sequence identity below 30% [28]. This confirms the model’s high degree of non-memorized generative capability, successfully exploring novel chemical space beyond native sequence trajectories.

In the evolutionary evaluation of sequence physicochemical features, the contrast goal of this study is not to assess the sequence design capability of RFdiffusion (which is essentially a pure backbone generation model), but to intuitively quantify the inherent information gap of the traditional two-stage “backbone-first, sequence-later” design paradigm when handling PPI tasks. As shown in Table 1, the pure geometric scaffolds output by the baseline model (occupied by poly-glycine) naturally lack side-chain physicochemical properties. In real-world drug design pipelines, such rigid geometric backbones devoid of sequence context often cause subsequent sequence design tools to fall into local optima. In contrast, full-sample statistics demonstrated that PPI-Diff, through end-to-end synergistic generation, simultaneously satisfied both geometric and physicochemical constraints in a single forward inference. The aromaticity of its generated sequences significantly jumped to 0.114, and the GRAVY hydrophobicity index reached a mildly hydrophobic level with a GRAVY index of 0.183, suggesting an enhanced potential to participate in hydrophobic interactions and form π−π electron stacking networks at the binding hotspots (Figure 5C). Furthermore, residue substitution matrices based on physicochemical properties confirmed (Figure 5B) that high-probability sequence substitutions strictly followed biophysical and chemical equivalence classification principles. Their macroscopic isoelectric points and hydrophobicity probability density distributions highly overlapped with native ligands, proving that the model possesses excellent physicochemical fidelity in real physical environments.

**Figure 6:**
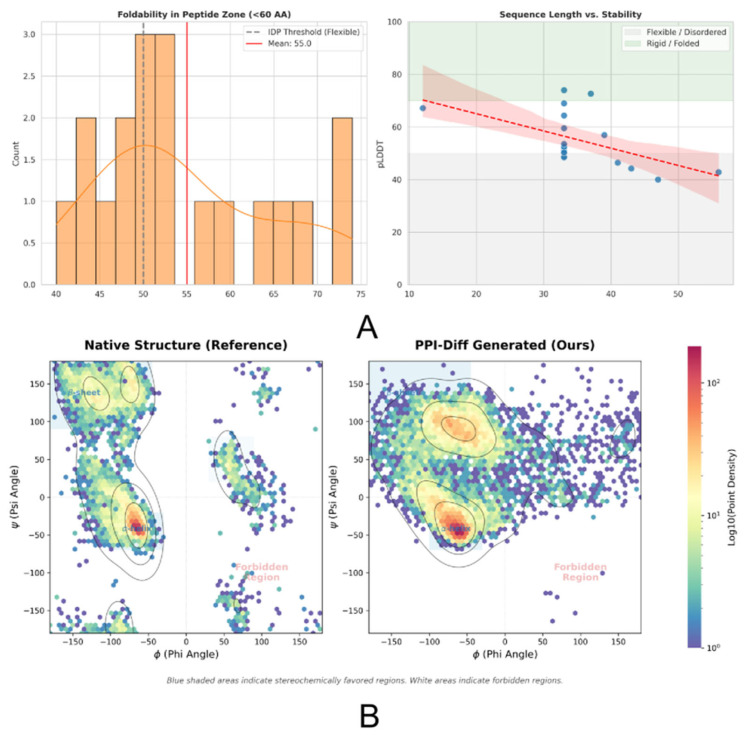
Synergistic validation of macroscopic conformational dynamics and microscopic stereochemistry. (**A**) Histogram of predicted confidence (pLDDT) distribution and its correlation with chain length. The mean value (55.0) indicates that the generated sequences exhibit moderate structural confidence aligning with the conformational flexibility required for binding. (**B**) Ramachandran density plot of the generated structures. The distribution points completely avoid stereochemically forbidden regions, verifying the physical rigor of the underlying NeRF geometric reconstruction module. In panel (**A**), the distribution illustrates the structural confidence of the generated sequences. In panel (**B**), the Ramachandran density plot displays the backbone dihedral angles, where the dense overlapping areas indicate the low-potential-energy core regions. The background contours/colors (e.g., darker shading) delineate the stereochemically allowed zones versus the forbidden zones with extreme steric hindrance.

In the macroscopic conformational stability evaluation, the mean pLDDT score distribution of the PPI-Diff generated sequences was 55.0 (Figure 6A), indicating a moderate level of structural confidence that is frequently associated with the inherent flexibility of intrinsically disordered regions. Short peptides exhibited a strong tendency to form stable local secondary structures, while long peptides significantly displayed random coil characteristics, once again corroborating the mechanism of peptide-target binding. At the microscopic backbone dihedral angle level, the Ramachandran density plot (Figure 6B) indicated that the distribution lattice of the generated conformations was highly focused in the low-potential-energy core regions permitted by stereochemical energy, effectively evading stereochemically forbidden zones with extreme steric hindrance [34]. This confirms that the differentiable geometric reconstruction module successfully and strongly constrained spatial interaction laws, ensuring that the highly flexible peptide macromolecules possess a high degree of realizability at the microscopic physical level.

It is worth noting that the enhanced conformational flexibility exhibited by PPI-Diff during peptide generation is partly due to the release of spatial degrees of freedom by the Riemannian manifold diffusion architecture. Objectively, it also reflects differences in training data distributions, as the model internalized the flexible interaction features of native PPI interfaces. This representation of the intrinsic flexibility of peptides physically aligns with the “fly-casting” mechanism and the “anchor and refine” dynamic binding mechanism characteristic of intrinsically disordered proteins (IDPs) [35].

### 3.3. Ablation Study and Loss Dynamics Analysis

To strictly quantify the independent contributions of the innovative components within the PPI-Diff framework to generative performance, this study conducted ablation experiments by stripping away core modules (Table 2). The quantitative data profoundly revealed the computational value of each mechanism. First, bidirectional physical symmetry enhancement is a crucial element in maintaining the network’s generalization capability. After removing this mechanism (w/o Bidirectional), the total loss surged from 0.1770 to 0.5127, and both structural and sequence losses showed significant deterioration. This indicates that unidirectional mapping easily leads to severe overfitting to specific topologies, whereas bidirectional enhancement forces the network to internalize universal interface symmetry rules. Second, ESM-2 semantic features acted as a key energy landscape navigation map. Removing this module (w/o ESM) caused the sequence cross-entropy loss to nearly double (to 0.2575), suggesting that cross-modal evolutionary semantics serves as a latent mapping mechanism that constrains the sequence distribution within physically viable regions; its absence leads to a decoupling of geometric requirements from evolutionary preferences, which is indirectly manifested as a noticeable decline in peptide physicochemical properties. Finally, the absence of resolution-aware gating (w/o Resolution-Aware) led to a distinct rebound in total loss (0.2641), suggesting that this mechanism successfully served as a data-adaptive frequency-domain filter, effectively shielding the spatial distortion interference caused by low-quality resolved samples and ensuring the precise convergence of microscopic geometric topologies.

### 3.4. Computational Efficiency and Inference Speed

To address the computational overhead concerns inherent in structure-based diffusion models, we benchmarked the inference efficiency of PPI-Diff. Operating entirely within the compact internal coordinate space (ϕ,ψ,ω angles) utilizing a lightweight 4-layer architecture, PPI-Diff demonstrates significant computational efficiency. As shown in Table 3, generating a peptide binder across targets of varying lengths (100 to 300 residues) consistently requires only ~5 s of inference time and marginal GPU VRAM (~24 MB) on a standard GPU. In contrast, traditional Cartesian-based graph diffusion models like RFdiffusion, which rely on heavy SE(3)-equivariant operations and extensive 3D graph updates over 1000 steps, typically demand minutes of inference time (>150 s) and gigabytes of VRAM (>6 GB) for similar tasks. This confirms that PPI-Diff successfully overcomes the computational bottlenecks of structure-based de novo design, offering a practical and scalable solution for high-throughput peptide generation.

### 3.5. Failure Case Analysis and Applicability Boundaries

**Figure 7:**
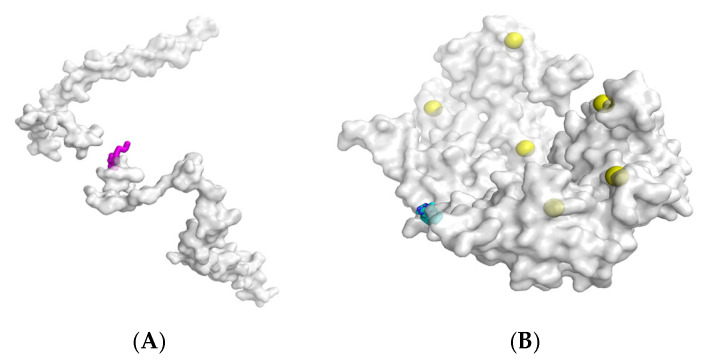
Typical failure cases and applicability boundaries of PPI-Diff. (**A**) On the extremely flat interface of O14949 (PDB: 5XTE), the generated peptide (magenta) lacks geometric constraints and anchor points, collapsing into a highly stretched, disorganized random coil on the surface. (**B**) For the co-factor-dependent target P06702 (PDB: 6DS2), the model fails to perceive the critical metal ions (yellow spheres). Consequently, the generated peptide is “blind” to the authentic coordination pocket, resulting in a misguided binding trajectory that completely misses the native interaction interface.The blue spheres at the bottom left represent this misguided generated peptide bound to an incorrect surface region.

Although PPI-Diff demonstrates significant quantitative advantages in modeling dynamic conformational flexibility, an objective analysis of its failure cases reveals the inherent physical boundaries of the current generative paradigm. We have identified two primary scenarios where the model’s generative quality degrades (illustrated in Figure 7).

(1) Ultra-flat target interfaces lacking geometric anchor points: On targets devoid of concave grooves or well-defined structural pockets, PPI-Diff occasionally struggles to stably anchor the binder. Because the model’s architecture inherently favors the release of conformational degrees of freedom—often generating flexible, IDP-like structures—the absence of geometric constraints on flat surfaces can cause the generated peptide to collapse into an extended, disordered random coil with insufficient interfacial contacts. This suggests that PPI-Diff operates most effectively on targets exhibiting moderate topological complexity.
(2) Cofactor- and solvent-dependent interaction interfaces: The current feature extraction module, encompassing ESM-2 and geometric convolutions, is strictly limited to canonical amino acid graphs. Consequently, the model remains insensitive to non-protein heteroatoms. For target interfaces where physical binding obligatorily relies on explicit water-mediated hydrogen bond networks or transition metal-ion coordination (e.g., zinc-finger domains), PPI-Diff fails to perceive these critical intermediate force fields. This “blind spot” leads to either steric clashes or aberrant binding trajectories. Future iterations must integrate explicit solvent and heteroatom embeddings to overcome these limitations.

## 4. Conclusions

This study addresses theoretical challenges in de novo peptide drug design, including rigid-body assumption conflicts, data resolution heterogeneity, and sequence-structure decoupling, by proposing PPI-Diff, a geometric diffusion framework that integrates physical awareness and evolutionary semantics. By discarding the rigid coordinate constraints of Euclidean absolute space and defining the denoising process on a dihedral Riemannian manifold, this architecture revises the deep generative paradigm from its underlying mechanics, endowing peptide chains with thermodynamic-level conformational degrees of freedom. In systematic validations, PPI-Diff effectively mitigated the tendency of baseline models to generate rigid straight-helix conformations. Future research will focus on constructing controlled-distribution experiments to isolate the independent contributions of architectural innovation and data distribution bias. Concurrently, owing to the end-to-end injection of ESM-2 evolutionary semantics, the model implicitly achieved synergistic matching of side-chain physical volumes and charge properties while maximizing interface geometric contact density, realizing macroscopic conservation of physicochemical properties.

Although this framework demonstrates methodological viability in exploring “undruggable” PPI targets, as a purely in silico generative computational framework, the current evaluation metrics of this study are primarily focused on geometric complementarity and statistical physicochemical features, which cannot yet be directly equated to actual biological affinity (e.g., Kd values). Moreover, existing general generative paradigms still lack sufficient granularity in physical field representation when dealing with atypical interfaces involving extensive water-mediated interactions or metal ion coordination. In summary, the binder sequences generated by PPI-Diff provide a viable lead-compound library for subsequent drug discovery. Moving forward, explicitly coupling Molecular Dynamics (MD) energy minimization into the reverse denoising trajectory and integrating this computational engine with closed-loop wet-lab experimental systems, such as Surface Plasmon Resonance (SPR) and phage display, will be critical pathways to achieve the translation from computational design to physical therapeutics. Furthermore, for practical deployment, we strongly recommend that practitioners ensure input target files are accompanied by accurately assigned local resolution metadata (especially for highly heterogeneous Cryo-EM structures), as the resolution-aware gating mechanism relies heavily on this tensor to actively suppress geometric noise. For uniformly high-resolution targets (e.g., <1.5 Å), the gating mechanism will seamlessly act as a uniform scaling factor without manual intervention.

## Figures and Tables

**Table 1:** Quantitative feature comparison between PPI-Diff and RFdiffusion on the binder design task.

Evaluation Dimension	Core Metric	RFdiffusion (Baseline)	PPI-Diff (Ours)
Conformational Dynamics	Helicity (%)	82.20%	39.50%
	Rama_Helix	0.821	0.001
	Shape_Ratio	6.54	2.22
	*R*g (Å)	10.94	13.16
Interfacial Binding Force	Contacts	10.3	14.5
Physicochemical Consistency	Aromaticity	N/A (Poly-Gly)	0.114
	GRAVY	N/A (Poly-Gly)	0.183

N/A: Not applicable. The baseline model RFdiffusion generates poly-glycine backbones lacking side chains, making sequence-dependent physicochemical metrics unmeasurable.

**Table 2:** Loss performance comparison of different computational variants on the validation set.

Model Variant	Total Loss	Structure Loss	Sequence Loss
w/o Resolution-Aware	0.2641	0.0450	0.2191
w/o Bidirectional (Unidirectional)	0.5127	0.0512	0.4615
w/o ESM (No Semantic)	0.3085	0.0510	0.2575
PPI-Diff	0.1770	0.0417	0.1353

w/o: Abbreviation for ‘without’, indicating the removal of a specific computational module in the ablation study.

**Table 3:** Inference efficiency comparison between PPI-Diff and the baseline model.

Target Length	Peptide Length	PPI-Diff Inference Time	PPI-Diff Peak VRAM	RFdiffusion Inference Time (Baseline) *	RFdiffusion Peak VRAM (Baseline) *
100 aa	10 aa	~5.14 s	~24 MB	~110–150 s	~5.0–6.5 GB
200 aa	20 aa	~5.04 s	~24 MB	~160–200 s	~7.0–8.5 GB
300 aa	30 aa	~5.21 s	~24 MB	~240–300 s	~9.0–12.0 GB

* Note: RFdiffusion baseline metrics are estimated based on standard 1000-step SE(3) graph diffusion overheads reported in the literature under comparable GPU hardware conditions. aa: amino acids; s: seconds; MB: megabytes; GB: gigabytes.

## Data Availability

The original protein–protein interaction data presented in the study are openly available in the STRING database (v12.0) and the Protein Data Bank (PDB). The source code, pre-trained weights, and the datasets used for the PPI-Diff framework during this study have been uploaded to GitHub and are accessible via https://github.com/SijiaLi1996/PPI-Diff (version 1.0.0) (accessed on 25 February 2026).
